# Automated breast lesion localisation in microwave imaging employing simplified pulse coupled neural network

**DOI:** 10.1371/journal.pone.0271377

**Published:** 2022-07-21

**Authors:** Maitreyee Dey, Soumya Prakash Rana, Riccardo Loretoni, Michele Duranti, Lorenzo Sani, Alessandro Vispa, Giovanni Raspa, Mohammad Ghavami, Sandra Dudley, Gianluigi Tiberi

**Affiliations:** 1 School of Engineering, London South Bank University, London, United Kingdom; 2 Breast Unit, Foligno Hospital, Foligno, Italy; 3 Department of Diagnostic Imaging, Perugia Hospital, Perugia, Italy; 4 UBT - Umbria Bioengineering Technologies Srl, Perugia, Italy; Information Technology University, PAKISTAN

## Abstract

MammoWave is a microwave imaging device for breast lesion detection, employing two antennas which rotate azimuthally (horizontally) around the breast. The antennas operate in the 1-9 GHz band and are set in free space, i.e., pivotally, no matching liquid is required. Microwave images, subsequently obtained through the application of Huygens Principle, are intensity maps, representing the homogeneity of the dielectric properties of the breast tissues under test. In this paper, MammoWave is used to realise tissues dielectric differences and localise lesions by segmenting microwave images adaptively employing pulse coupled neural network (PCNN). Subsequently, a non-parametric thresholding technique is modelled to differentiate between breasts having no radiological finding (NF) or benign (BF) and breasts with malignant finding (MF). Resultant findings verify that automated breast lesion localization with microwave imaging matches the gold standard achieving 81.82% sensitivity in MF detection. The proposed method is tested on microwave images acquired from a feasibility study performed in Foligno Hospital, Italy. This study is based on 61 breasts from 35 patients; performance may vary with larger number of datasets and will be subsequently investigated.

## Introduction

Mammography is the current gold standard breast-screening tool, although it has been demonstrated to be less effective for dense breasts [1]. Studies have shown that screening can detect breast cancer at an earlier stage reducing treatment times, offering greater treatment choices, and therefore improving survival rates. However, ongoing debates remain i) due to possible risks related to ionising radiation exposure and ii) on the related optimization of the screening strategy [2]. Hence, the medical community and related stakeholders must seek alternative, accurate and safe mass-screening tools, which could be used without age and condition restrictions [3]. Due to the recent and continuous advancement of radio-frequency hardware technology/sensors, microwave breast imaging has become a potential alternative or additional imaging method to mammography [4]. Microwave imaging is generally divided into two major groups: radar-based imaging and microwave tomography. Radar-based imaging methods perform a linear reconstruction of the image, which is a qualitative scattering map in arbitrary units. Microwave tomography is based on inverse scattering algorithms, which lead to quantitative maps of permittivity and conductivity; however, inverse scattering algorithms may be affected by mathematical instability. Both radar-based imaging and microwave tomography still have lower spatial resolution than mammography. Microwave imaging techniques exploit the dielectric properties contrast between the healthy tissues and tissues with lesions [5–9]; specifically, a contrast up to 5 has been reported between healthy fatty tissue and malignant tissue, while it decreases between healthy fibroglandular and malignant tissues [10].

Artificial intelligence (AI) and machine learning (ML) based breast lesion detection have been a focus by researchers for assisting medical practitioners in their diagnostics process. With the help of appropriate data sets, AI and ML technology can also be highly useful in the future lesion prediction purpose [11]. Techniques implementing AI and ML acquire the normal and abnormal patterns from the provided data (specific to the system under test) to form decisions based on the specific models’ learning capabilities [12–15]. A hybrid approach using fuzzy sets, wavelet transformation, pulse coupled neural network (PCNN), and support vector machine (SVM) for breast cancer classification has been presented in [16]. This hybrid approach was tested with MRI dataset. However, the accuracy of their method was limited while processing high dimension data, increases the computational complexity which makes it difficult for real-time operation and widespread use. Another PCNN based detection of breast’s micro-calcification for mammogram has been proposed in [17]. Here, the combination of Otsu thresholding, morphology, bi-orthogonal wavelet and PCNN was employed for automatic breast cancer detection. A combination of kinetic features and diffusion-weighted imaging (DWI) of morphology from MRI to discriminate malignant from benign lesions is presented in [18]. This method investigated the data collected from 234 patients and tested the method’s effectiveness via cross-validation.

A very limited number of microwave imaging and AI-based studies have been found to detect breast cancer, with most studies and investigations executed using either synthetic data [19] or measurements from phantoms [20]. MammoWave (UBT Srl, Italy) is one of the very few microwave breast-imaging prototypes constructed, tested, and validated at clinical level. MammoWave, which advantageously works in air with 2 antennas rotating in the azimuth plane and operating within the 1-9 GHz band, has an innovative frequency domain imaging algorithm based on Huygens Principle (HP) [21, 22]. An intelligent classification system to support clinicians has been proposed from MammoWave raw-data signals by the authors [23].

The proposed work employs MammoWave microwave images and demonstrates PCNN based adaptive breast image segmentation and lesion detection on the meta-analyses clinical data. In this work, we introduce a notable combination comprising a simplified PCNN model with statistical box plot based thresholding, investigating their ability to detect the location of the lesion within the breast. First, the relationship between each iterative step of PCNN and the segmented images is studied thoroughly to improve the detection rate where, without training, the lesion’s location and shape are captured (see Section: Adaptive Image Segmentation). Second, the breast images with and without findings have been studied separately to assess our method’s effectiveness, understanding benefits and limitations(see Section: Non-parametric Thresholding). The proposed method has been tested empirically on microwave images of 61 breasts. Each breast has its own correspondent output of the radiologist study review, which has been used as gold standard for classification of the breasts in two categories: breasts having no radiological finding (NF) or benign finding (BF), breasts with malignant finding (MF).

## Methods & materials

**Fig 1:**
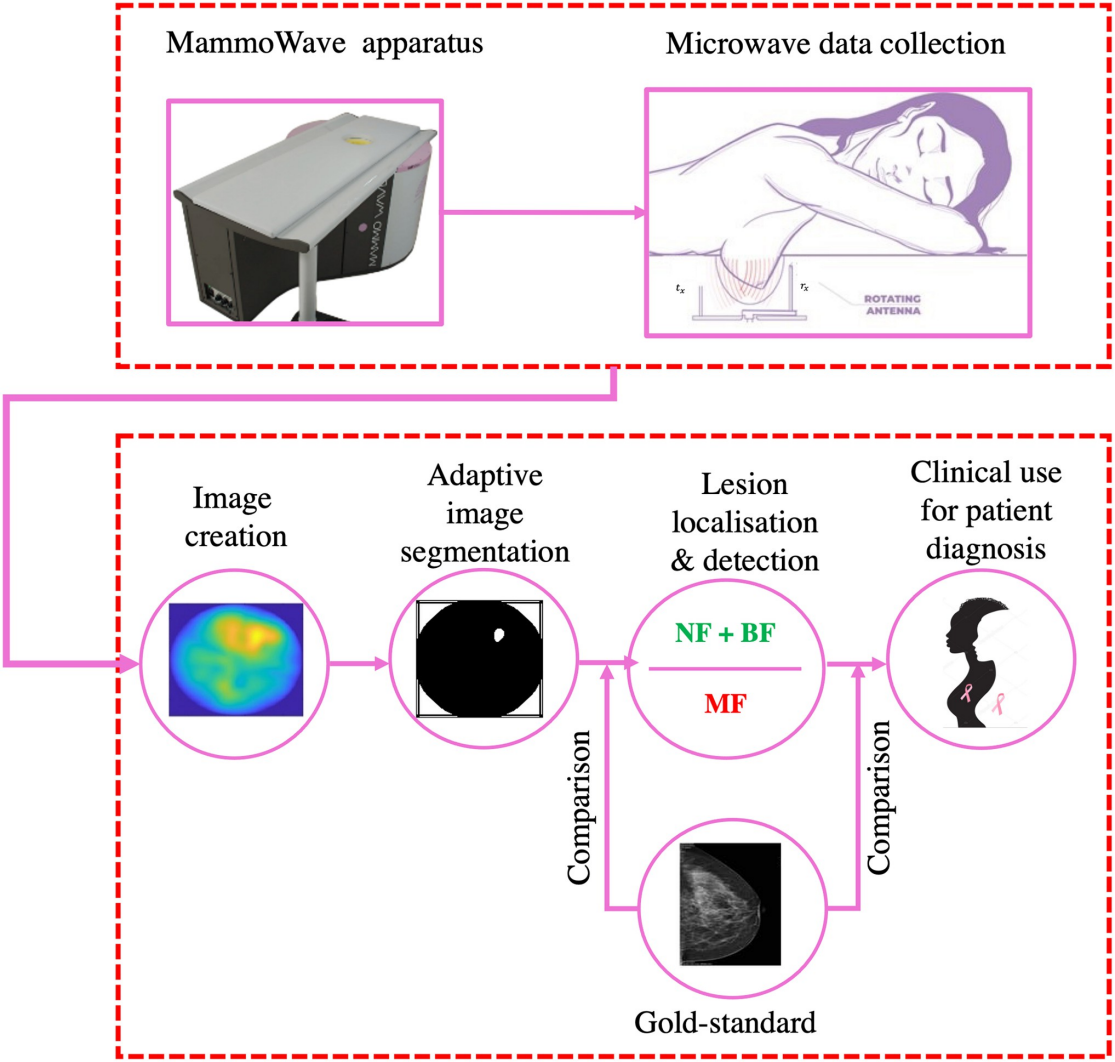
Flow chart of the proposed work; the clinical data collection procedure using MammoWave system and the proposed lesion localisation using the MammoWave created images.

The proposed method for breast lesion localisation has been conducted by following four primary steps: data acquisition from the MammoWave system, adaptive image segmentation, dynamic thresholding for breast classification, and validation with the gold-standard, and is shown in the Fig 1. Each step is described in the below subsections.

### Apparatus description and data details

The MammoWave apparatus (UBT Srl, Italy) is shown in Fig 1 and comprises an aluminum cylindrical hub with two antennas: one transmitting (*t*_*x*_) and one receiving (*r*_*x*_), transceive over the 1-9 GHz radio-frequency band. The hub contains a hole with a cup (polylactic acid (PLA) to ensure biocompatibility) where a patient can comfortably place their breast by lying in a horizontal position (as shown in Fig 1).

Specifically, three different sizes of cups are available for patient tests (i.e., with diameter 95 mm, 110 mm, 134 mm), and the clinical study coordinator chooses the most appropriate size to best fit the subject’s breast. The cup has a rim width of 1 mm; and it has been found that this thickness does not impact microwave imaging [24].

**Fig 2:**
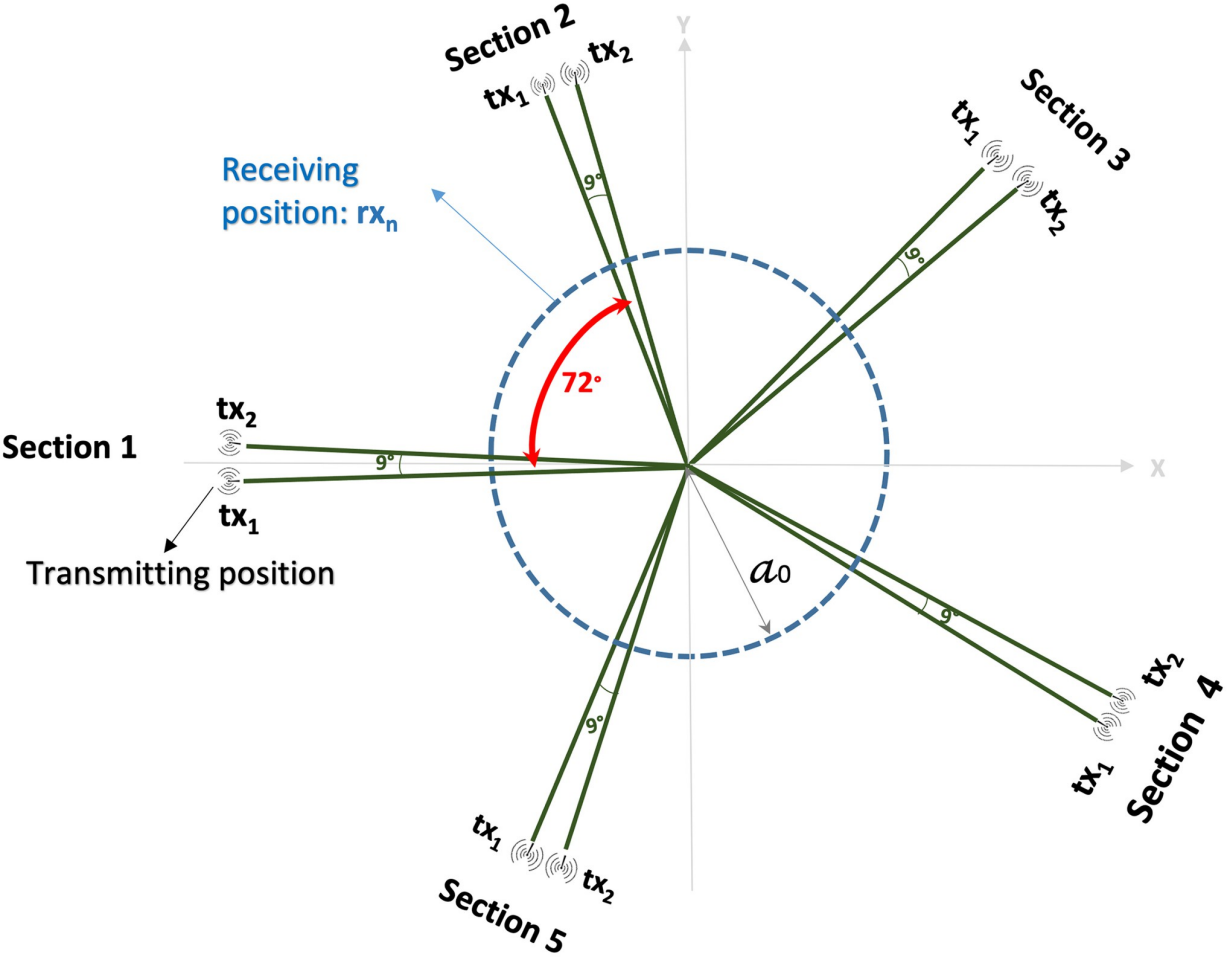
The apparatus measurement procedure; the antennas inside the container (covered to absorb microwaves) are fitted at the constant height, in free space and can rotate across the azimuth for collecting the microwave signals from diverse angular locations. For every transmitting and receiving spot, the complex *S*_21_ is gathered from 1 to 9 GHz, along with 5 MHz sampling.

Fig 2 provides details of the microwave acquisition. It is pivotal to note that no matching liquid is required in the apparatus, and unlike some current methods, no breast compression is needed at any point throughout the data acquisition. The antennas inside the container (covered to absorb microwaves) are fitted at the constant height, in free space and can rotate across the azimuth for collecting the microwave signals from diverse angular locations. The transmitting and receiving antennas are attached to a 2-port VNA (Cobalt C1209, Copper Mountain, Indianapolis, IN) that operates up to 9 GHz. Measurements have been accomplished by recording the complex scattering parameter *S*_21_ in a multi-bistatic fashion, i.e. for each transmitting position *tx*_*m*_ the receiving antenna is shifted to measure the received signal in each 4.5°, towards all together 80 receiving points *rx*_*n*_. All the measurements have been performed by using 10 transmitting position, displaced in 5 sections centered at 0°, 72°, 144°, 216°, and 288°. For every transmitting and receiving spot, the complex scattering parameter *S*_21_ is gathered from 1 to 9 GHz, along with 5 MHz sampling. In the current MammoWave configuration: the transmitting antenna is placed at a distance of 30 cm from the centre of the cup; the receiving antenna is placed at a distance of 7 cm from the centre of the cup, more details can be found in [24]. MammoWave acquisition time is approximately 10 minutes (per breast).

In-vivo validation of MammoWave on volunteers in Perugia Hospital and Foligno Hospital was approved in 2015 by the Ethical Committee of Umbria, Italy (N. 6845/15/AV/DM of 14/10/2015, N. 10352/17/NCAV of 16/03/2017, N 13203/18/NCAV of 17/04/2018). The protocol is responsible for a feasibility study of the proposed apparatus to detect breast lesions, with the intention of quantifying its accuracy. The inclusion criteria allowed female volunteers with intact breast and with a radiologist study output obtained through conventional exams (mammography and/or ultrasound and/or magnetic resonance imaging). All protocols and procedures were in accordance with both institutional and national ethical standards in research, and World Medical Association Declaration of Helsinki (1964) and its future amendments or analogous ethical standards. Prior to the trial, all participants have been requested to read and sign both the informative sheet and informed consent form.

This study comprises images and data of 61 breasts from 35 patients; a summary of patient information is tabulated in Table 1. Each breast has its own corresponding radiologist study review output, used as gold standard for breast classification. In greater details, the radiologist study review initially classified breasts into the categories: NF breasts; WF breasts. Next, a one-year regular clinical or histological follow up provided the final assessment of the breast’s radiological findings, i.e. lesions: malignant or benign. Additional details of the radiologist study review have been also collected and are tabulated in Table 2.

**Table 1:** Summary of patient’s information used in this study.

Total patients	35
Total subjects (breasts)	61
Average patient’s age (in year)	52
Number of patients age between 20-49 year	23
Number of patients age between 50-80 year	38

**Table 2:** Breast index, patient index, age, examined breast (L /R), breast category (NF, BF, and MF) from the radiologist (gold standard information), lesion position, and dimensions are all listed here.

Breast Index	Patient Index	Patient Age	Breast Side (L/R)	Breast Type	Inclusion Position	Inclusion Dimension (Maximum Axis)	Final assessment (Benign-BF/ Malignant-MF)
1	1	48	L	WF	Upper Zone	Not Available	Benign
2	1	48	R	NF	–	–	–
3	2	65	L	WF	Upper Zone	Not Available	Benign
4	2	65	R	NF	–	–	–
5	3	65	R	NF	–	–	–
6	4	57	R	NF	–	–	Benign
7	4	57	L	NF	–	–	–
8	5	40	L	WF	More Areas	Not Available	Benign
9	5	40	R	WF	Upper Zone	9 Mm	Benign
10	6	52	L	WF	Upper Zone	Not Available	Malignant
11	6	52	R	NF	–	–	–
12	7	36	L	NF	–	–	–
13	8	47	L	WF	More Areas	Multi Inclusions of 1mm	Benign
14	9	54	R	NF	–	–	–
15	9	54	L	NF	–	–	–
16	10	55	R	WF	Upper Zone	1, 6 Mm	Benign
17	10	55	L	WF	Upper Zone	3, 8 Mm	Benign
18	11	51	L	WF	Upper Zone	Not Available	Benign
19	12	54	R	WF	Upper Zone	Not Available	Benign
20	12	54	L	NF	–	–	–
21	13	77	R	WF	Upper Zone	17 Mm	Malignant
22	14	61	R	WF	Upper Zone	15 Mm	Malignant
23	14	61	L	WF	Upper Zone	Not Available	Benign
24	15	50	R	NF	–	–	–
25	15	50	L	WF	Lower Zone	10 Mm	Benign
26	16	67	L	WF	Lower Zone	5, 5 Mm	Malignant
27	16	67	R	NF	–	–	–
28	17	49	L	WF	Upper Zone	Not Available	Benign
29	17	49	R	NF	–	–	–
30	18	70	L	WF	Upper Zone	Not Available	Malignant
31	19	42	L	WF	Upper Zone	7 Mm	Benign
32	20	67	L	WF	Upper Zone	10 Mm	Benign
33	20	67	R	NF	–	–	–
34	21	56	R	WF	Upper Zone	31 Mm	Malignant
35	22	43	R	WF	Upper Zone	12 Mm	Benign
36	22	43	L	NF	–	–	–
37	23	51	L	WF	Lower Zone	Not Available	Benign
38	23	51	R	NF	–	–	–
39	24	59	L	WF	Upper Zone	11 Mm	Malignant
40	24	59	R	NF	–	–	–
41	25	40	L	WF	Lower Zone	32 Mm	Benign
42	25	40	R	NF	–	–	–
43	26	35	R	WF	Upper Zone	7 Mm	Benign
44	26	35	L	NF	–	–	–
45	27	37	L	WF	Lower Zone	25 Mm	Benign
46	27	37	R	NF	–	–	–
47	28	43	R	WF	Upper Zone	Not Available	Malignant
48	28	43	L	NF	–	–	–
49	29	54	R	WF	Upper Zone	18 Mm	Benign
50	30	49	L	WF	Upper Zone	16 Mm	Benign
51	30	49	R	NF	–	–	–
52	31	56	L	WF	Upper Zone	27 Mm	Malignant
53	31	56	R	NF	–	–	–
54	32	63	L	WF	Upper Zone	6 Mm	Malignant
55	32	63	R	NF	–	–	–
56	33	55	R	WF	Upper Zone	23 Mm	Malignant
57	33	55	L	WF	–	Not Available	Benign
58	34	64	R	WF	Upper Zone	Not Available	Benign
59	34	64	L	NF	–	–	–
60	35	37	R	WF	Lower Zone	15.4mm	Benign
61	35	37	L	WF	Not Available	Not Available	Benign

### Image formation procedure

Measurements are performed by recording the complex scattering parameter *S*_21_ in a multi-bistatic fashion. Specifically, *rx* can rotate to measure the received signal at the points displaced on a circular surface having radius *a*_0_. The received signals can be expressed as ${S_{21}}_{n}^{m,p}\left(a_{0},\phi_{n};tx_{m,p};f\right)$, with *n* = 1, 2, …, *N*_*PT*_, where *N*_*PT*_ denotes the total number of receiving points, 80 in this case; *m* = 1, 2 …, 5 indicates the transmitting sections, *p* = 1, 2 indicates the position inside each transmitting section; and *f* is the frequency. The Huygens principle (HP) has been employed on the received signals to calculate the field inside the cylinder. An image is then generated from this field, which is a homogeneity map of the dielectric properties. To remove the artefacts, the subtraction between *S*_21_ from two measurements belonging to the doublet of the same section has been employed. The below equation shows the HP based procedure in Eq (1).

$$\begin{array}{c} E_{HP,2D}^{rcstr}\left(\rho ,\phi ;tx_{m,p}-tx_{m,p^{\prime }};f\right)\propto \sum_{n=1}^{N_{PT}}\left({S_{21}}_{n}^{m,p}\left(a_{0},\phi_{n};tx_{m,p};f\right)-{S_{21}}_{n}^{m,p^{\prime }}\left(a_{0},\phi_{n};tx_{m,p};f\right)\right)G(k_{1}|\vec{\rho_{n}}-\vec{\rho }\left|\right) \end{array}$$ (1)

where $\left(\rho ,\phi \right)\equiv \vec{\rho }$ is the observation point, Δ_*s*_ is the spatial sampling. *k*_1_ is the wave number, and *G* is the Green’s function. The “reconstructed” internal field has been specified by the string *rcstr*. Note, if the conductivity of the media is not equal to zero, Eq (1) compensates the attenuation experienced while going into the media. Let, *N*_*F*_ frequencies has been used *f*_*i*_ in the band *B*, it shows that the intensity of the image *I* achieved via following equation, i.e. by summing incoherently all the solutions of all the sections:

$$\begin{array}{c} I\left(\rho ,\phi \right)=\sum_{m=1}^{5}\sum_{p=1}^{2}\sum_{i=1}^{N_{F}}{\left|E_{HP,2D}^{rcstr}\left(\rho ,\phi ;tx_{m,p}-tx_{m,p^{\prime }};f_{i}\right)\right|}^{2} \end{array}$$ (2)

The two-dimensional (2D) image is created by Eq (2) in the azimuthal, i.e. coronal plane. Because the receiving antenna is in free space, the images have been obtained using the free space dielectric constant in Eq (1). Regarding the conductivity (denoted with *σ*) for each breast, multiple different microwave images may be produced, i.e., a conductivity weighing has been applied by varying the conductivity in Eq (1). In this paper, four different conductivity images have been investigated denoted as *σ*_1_ = 0.01 *S*/*m*, *σ*_2_ = 0.20 *S*/*m*, *σ*_3_ = 0.40 *S*/*m*, *σ*_4_ = 0.60 *S*/*m*; the outcomes were analysed to determine the optimal image. It should be highlighted that the conductivity values used here are in agreement with the breast conductivity average values reported in [25].

**Fig 3:**
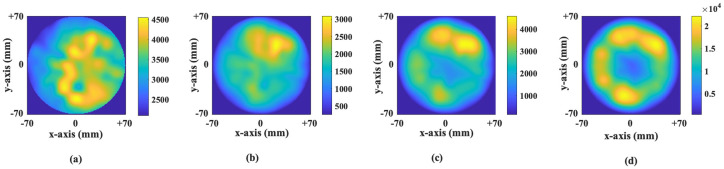
Microwave images of a single breast for different conductivity level generated via the MammoWave signal. (a) represent the image created for *σ*_1_ conductivity level, (b) represent the image created for *σ*_2_ conductivity, (c) represent the image created for *σ*_3_ conductivity, and (d) represent the image created for *σ*_4_ conductivity. Images given here are two-dimensional (2D) image in the azimuthal, i.e. coronal, plane. The x-axis and y-axis are given in meter and the colour bar represents the intensity in arbitrary units.

Images generated by the MammoWave are intensity maps, given in linear arbitrary units, representing the homogeneity of tissues’ dielectric properties. Microwave images have been first obtained in a cylindrical grid with 7*cm* radius (which matches to the radius of the receiving antenna), a radial sampling of 1*mm* and an azimuthal sampling of 3°. Furthermore, all images have been interpolated on a 2D Cartesian grid having *X* and *Y* sampling of 1*mm*. The images have been created using free space dielectric constant because of the presence of receiving antenna in free space. It has been verified via extensive phantoms experiments that this choice allows detection [26, 27], even if it introduces a slight error in localisation [24, 28]. An example of the produced images for the different conductivity shown in Fig 3, where Fig 3(a)–3(d) represents for the *σ*_1_, *σ*_2_, *σ*_3_, and *σ*_4_ conductivity images respectively created from the identical breast signal.

## Adaptive image segmentation

Once the microwave image is obtained, image processing on the resulting image is subsequently performed. A neural-network model Pulse Coupled Neural Network (PCNN) is employed to perform the adaptive image segmentation to detect the prominent region of the breast images. This neural network is a third generation, single-layer, two-dimensional neural network based on cat visual cortex properties and further modified for adaptive segmentation of the MammoWave images to create segmented binary images by effective simulation of a synchronous behaviour [29, 30]. This comprises three parts: an input part, a modulation part, and a pulse generator part. In the input part, each neuron receives signals through feeding (*F*_*i*,*j*_) and linking (*L*_*i*,*j*_) channels, where i, j stands for position of the neurons of the image in nth iteration. Feeding consists of external sources (*S*_*i*,*j*_) which is the normalised pixel value of the input image. Linking represents, by the constant synaptic weights from neuron (*W*_*i*,*j*,*k*,*l*_) and is linked with its specified neighbouring neurons, referred to as *N*_*i*,*j*_, where *k*, *l* indicate the location of the neighbour neuron of *i*, *j*. The modulation part is the non-linear combination of both the feeding and linking signals through the linking coefficient *β*, as the internal activity (*U*_*i*,*j*_). Finally, the pulse generator part produces a pulse to fire neurons using an adaptive threshold variable (*T*_*i*,*j*_) as a step function to control the output neuron firing event. It works in such a way that when the threshold decreases exponentially this causes more firing neurons in the subsequent step. After thresholding, a pulse output *O*_*i*,*j*_ is produced based on the mathematical models shown below in Eqs (3)–(7), where *α*_*T*_ is the threshold decay time constant and *V*_*T*_ is the threshold normalisation constant.

$$\begin{array}{c} F_{i,j}\left[n\right]=S_{i,j} \end{array}$$ (3)

$$\begin{array}{c} L_{i,j}\left[n\right]=\sum_{k,j\in N_{i,j}}W_{i,j,k,l}O_{k,l}\left[n-1\right] \end{array}$$ (4)

$$\begin{array}{c} U_{i,j}\left[n\right]=F_{i,j}\left[n\right]\left(1+\beta L_{i,j}\left[n\right]\right) \end{array}$$ (5)

$$\begin{array}{c} O_{i,j}\left[n\right]={\{}_{0\;\;\;\;Otherwise}^{1\;ifU_{i,j}\left[n\right]>T_{i,j}\left[n-1\right]} \end{array}$$ (6)

$$\begin{array}{c} T_{i,j}\left[n\right]=e^{-\alpha_{T}}T_{i,j}\left[n+1\right]+V_{T}O_{i,j}\left[n-1\right] \end{array}$$ (7)

**Fig 4:**
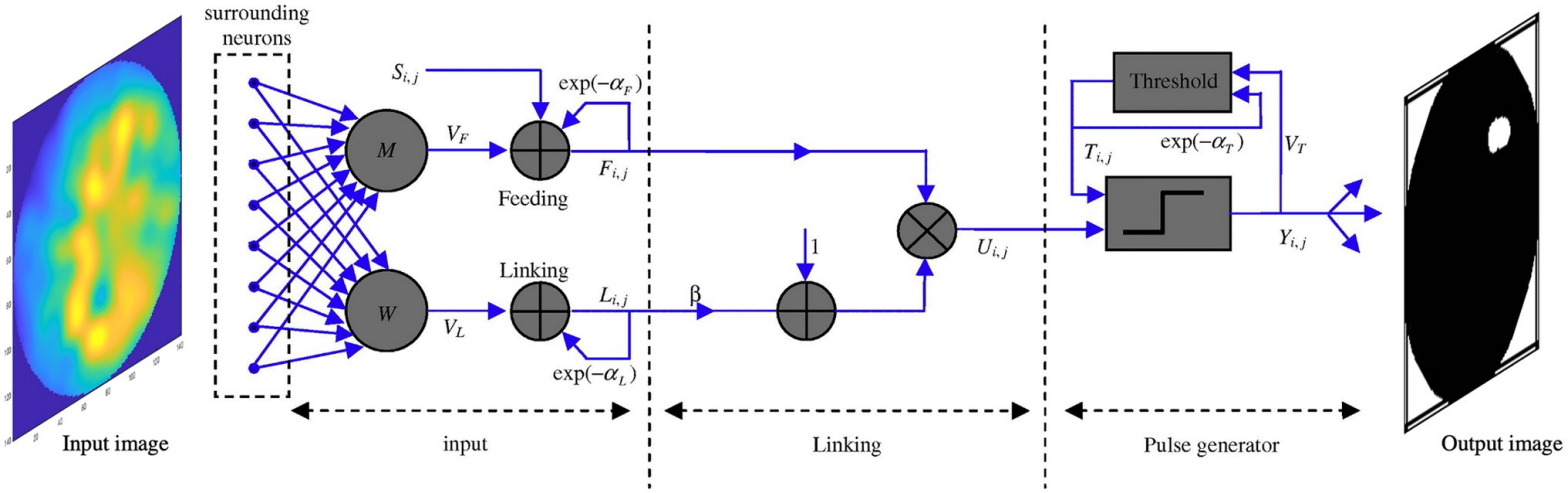
The flow graph of the adaptive image segmentation using PCNN [31].

The working principle of PCNN image segmentation graphically displayed in Fig 4, where input images are fed into the PCNN to obtain binary output image and detect lesion region.

## Non-parametric thresholding

Further, the box and whisker diagram [32] has been incorporated with the thresholding approach to resolve the risk level of the breast pattern detection. This is a non-parametric statistical approach, which graphically depicts the quantitative NF+BF and MF image data through their quartiles. The upper (*Q*_3_) and lower (*Q*_1_) limit of the boxes indicate the 75^th^ and 25^th^ percentiles of the relative dielectric property’s variability respectively, whereas the middle-line (*Q*_2_) indicates the data median. Hence, the quartiles have been experimented and found *Q*_1_ and *Q*_3_ both quartiles contain approximately similar responses for both the healthy and unhealthy breasts. Thus, a 2D Gaussian smoothing kernel has been applied to the image and then average the *Q*_3_ value for both of the breast type and found that average *Q*_3_ as the significant threshold value to detect the breast type from these dataset.

## Experimental result analysis & discussion

This work has four major steps: parameter selection, identification of a lesion through adaptive segmentation, decision making and findings of NF+BF and MF breast, and a performance measure of the proposed study.

As the PCNN results rely on the found parameters, it has been chosen by successive estimation [31, 33]. The parameters are the threshold decay constant (*α*_*T*_), normalization constant (*V*_*T*_), and linking strength (*β*) denoted in Section: Adaptive Image Segmentation. In practice, users need to adjust the parameter value according to their requirements. Practically *V*_*T*_ should be set to a high value, if it is set to low value causes more frequent firing neurons, which loses the information of granular changes over the iterations. This produces over-segmented images because the region could not grow fully. On the contrary, *β* and *α*_*T*_ values should be set to low value, it they are set to high leading to under-segmentation, which will ignore the small changes between each iteration and output cannot be measured or realised in detail.

Thu, the value of *V*_*T*_ is set as ‘20’ as a high value avoids the neurons firing instantly. Experimentally the value of *β* and *α*_*T*_ are set to ‘3’ and ‘0.2’ respectively. Appropriate parameter selection is paramount to a successful low computational complexity detection algorithm for accurate lesion location. Therefore, the quantitative analysis of each parameter has been considered to achieve optimum performance by the network towards true highlighted cell location detection.

### Lesion localisation from PCNN

Breast images are processed through PCNN, producing a binary output after every iteration. Each iteration creates different segmented images, where in the first iteration all the neurons compare their input pixel value with the defined threshold parameter (*V*_*T*_) initialising the firing of all the neurons. In the second iteration, the threshold decay constant (*α*_*T*_) reduces the threshold and fewer neurons are subsequently fired. Likewise, in every iteration the decay parameter continue to decrease the threshold, thus fewer and more intensity neurons are fired producing finer output images after each iteration. This repeats until all the neurons intensity become higher than the threshold since the threshold decreases after every iteration. This method does not have an auto-stop criterion thus the stopping condition is selected experimentally and is the 32^nd^ iteration in this proposed work as after that its overfitted and keep on generates similar output images.

**Fig 5:**
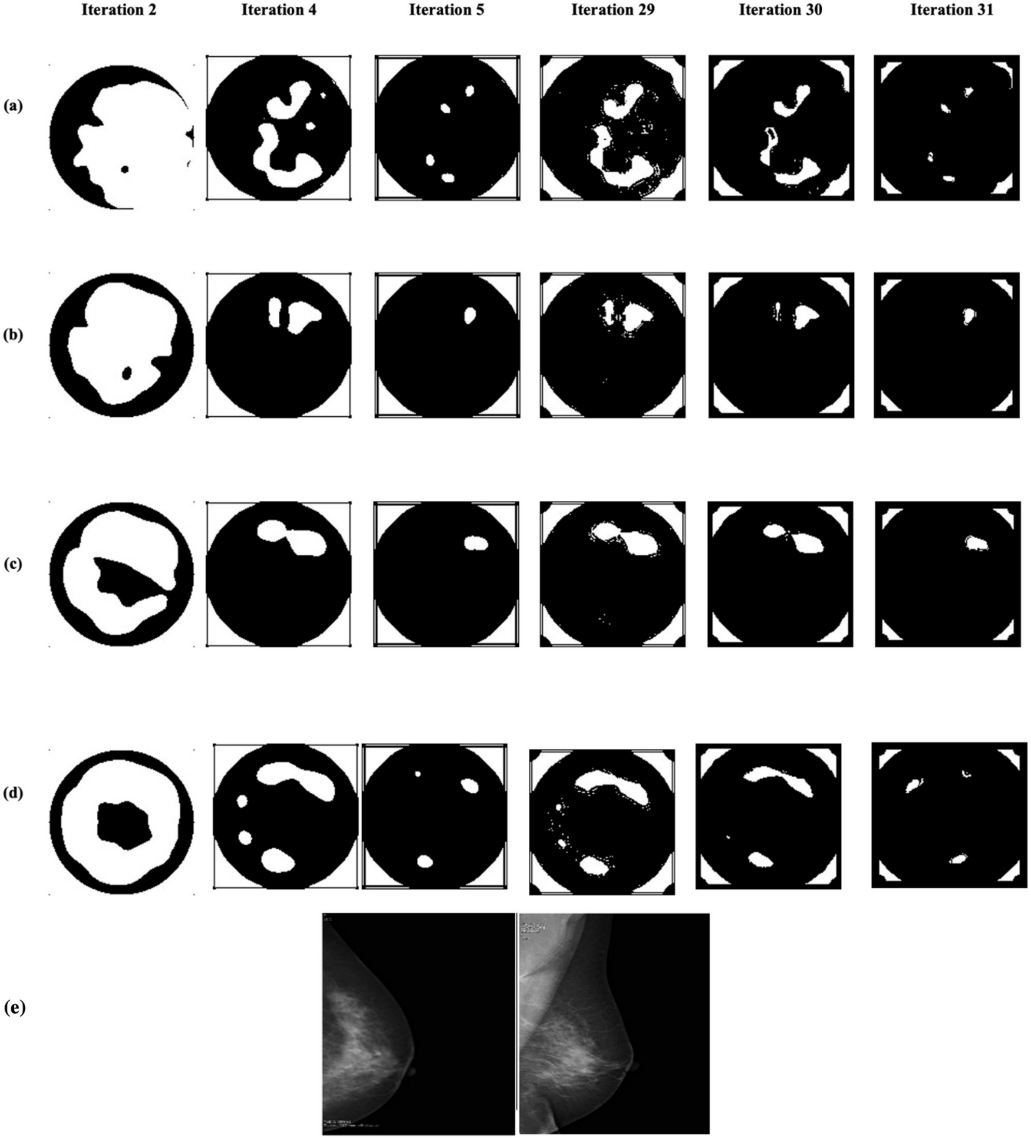
Six PCNN iterations for one MF breast images for different conductivity (a) input image formed using *σ*_1_, (b) input image formed using *σ*_2_, (c) input image formed using *σ*_3_, and (d) input image formed using *σ*_4_. The radiologist study review “MF” for this heterogeneously dense breast has been obtained with the support of mammography images given in the bottom row, giving as output the presence of a cluster of microcalcifications, plus follow-up.

Fig 5 displays the results obtained from six PCNN iterations for one MF microwave breast image for different conductivity results, each row represents four different conductivity level (*σ*_1_, *σ*_2_, *σ*_3_, and *σ*_4_) images (explained in Section: Image Formation Procedure) and each column represents the different iterations. In Iteration-2 more neurons have fired for all the levels, whereas the number of firing neurons reduces significantly at Iteration 4. This process continues until Iteration-32 and shows similar patterns keep on segmented adaptively because of the auto-wave nature of the neural network. It is observed that the patterns generated by Iteration-4 are similar to Iteration-29 and 31, whereas the Iteration-5 result is always similar to Iteration-31. The radiologist study review “MF” for this heterogeneously dense breast has been obtained with the support of mammography images given in the bottom row, generating as its output the presence of a cluster of microcalcifications, plus the follow-up. It has been observed that the adaptiveness of the threshold selection is highly accurate with the pixel’s intensity variation and in particular, optimal iteration found as the Iteration-5.

**Fig 6:**
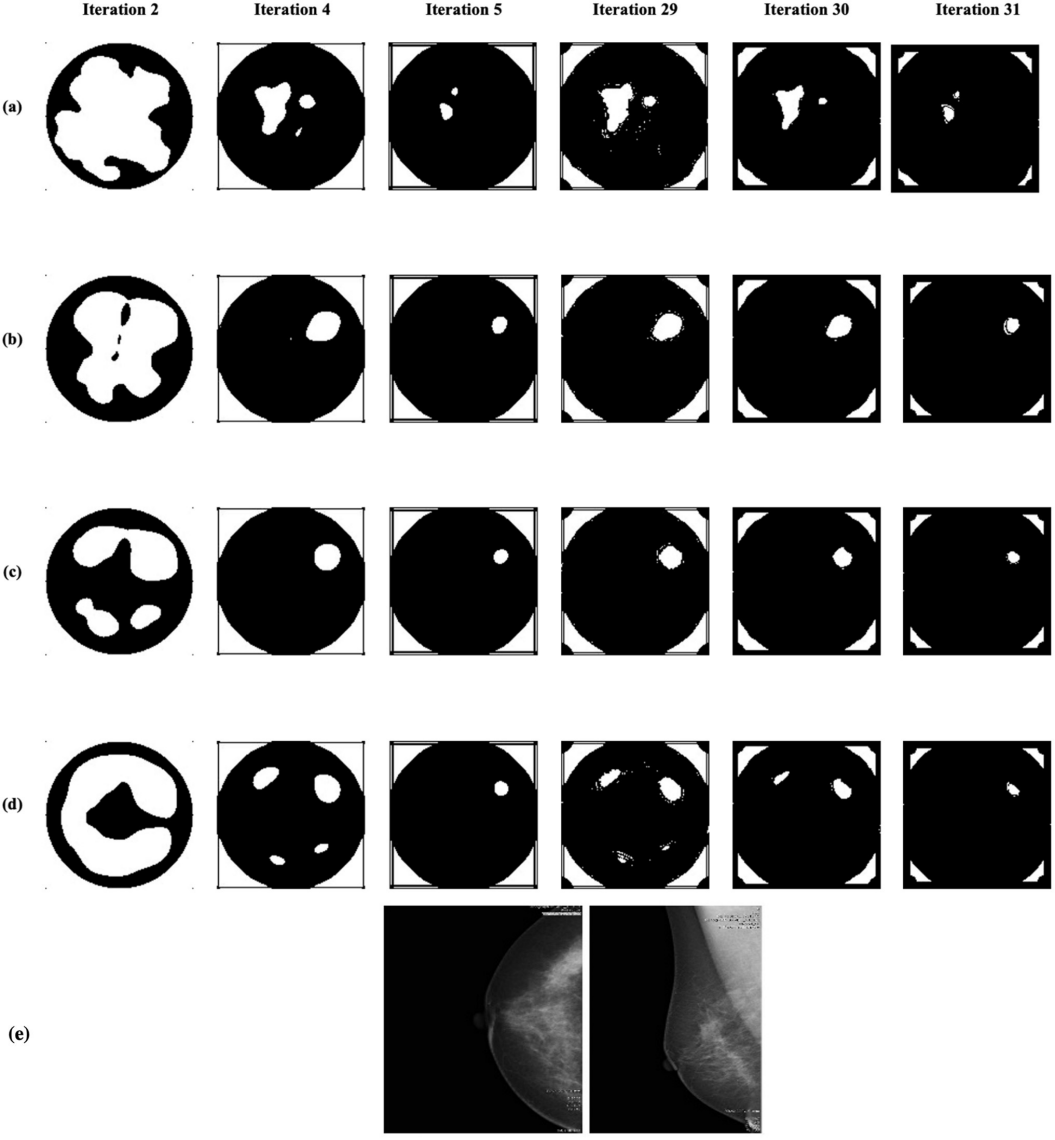
The results obtained over PCNN iterations for one of the NF+BF breast images with different conductivity: (a) Input image formed using *σ*_1_, (b) input image formed using *σ*_2_, (c) input image formed using *σ*_3_, and (d) input image formed using *σ*_4_. The radiologist study review “NF” for this scattered area of fibroglandular density breast has been obtained with the support of mammography images given in the bottom row.

Fig 6 shows the segmented binary images of a healthy breast (that comes under the category of NF+BF breast) patterns for the four different conductivity levels. It has been observed that the patterns generated by Iteration-4 is alike with Iteration-29 and 31, whereas Iteration-5 result is similar to Iteration-31. Thus, the Iteration-5 is also found to produce the best segmented images amongst all other iterations. Thus, five iterations are considered for localising the suspected tissues within the images using PCNN. The correspondent mammographic images are also given below in Fig 6.

Concerning microwave images, the optimal conductivity level has been experimentally analysed along with the optimal iteration number. In Figs 5 and 6, the outputs of column 3 (Iteration-5) for every row shows a significant difference in locating the abnormal tissues. Figs 5(c) and 6(c) indicates only clearly detected regions (using *σ*_3_) whereas all other segmented images (where the input images are generated using *σ*_1_, *σ*_2_, and *σ*_4_) show multiple, less significant/ redundant regions, prohibiting a definitive decision for healthy or unhealthy breast identification. The lesion localisation experiment has been robustly performed for all the patients’ breast images available in this study and Iteration-5 with the conductivity level *σ*_3_ was found to be the optimal combination to locate possible lesions. Such findings are in agreement with that reported in [34] where images’ parameters, i.e., features, of microwave images obtained using different conductivity levels have been introduced and used for breast lesions detection. The radiological reviews have also been compared here for WF breasts (from Table 2) to perceive if the identified lesion positions are or are not the same which gives a positive impression on the results obtained from PCNN. All the locations of the lesions found matches with the radiologist’s location findings.

### Malignant lesion detection from non-parametric thresholding

**Fig 7:**
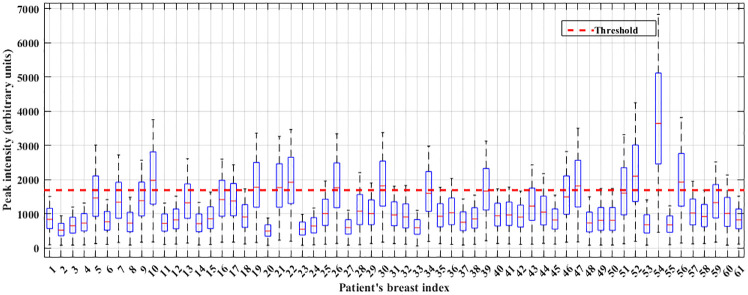
Box whisker plot for the examined breast images with the thresholding. 61 breasts’ data have been filtered through Gaussian kernel to decide the threshold value, where the *x*-axis represent the number of breasts’ index and *y*-axis represent the peak intensity (arbitrary units) of each breast.

**Fig 8:**
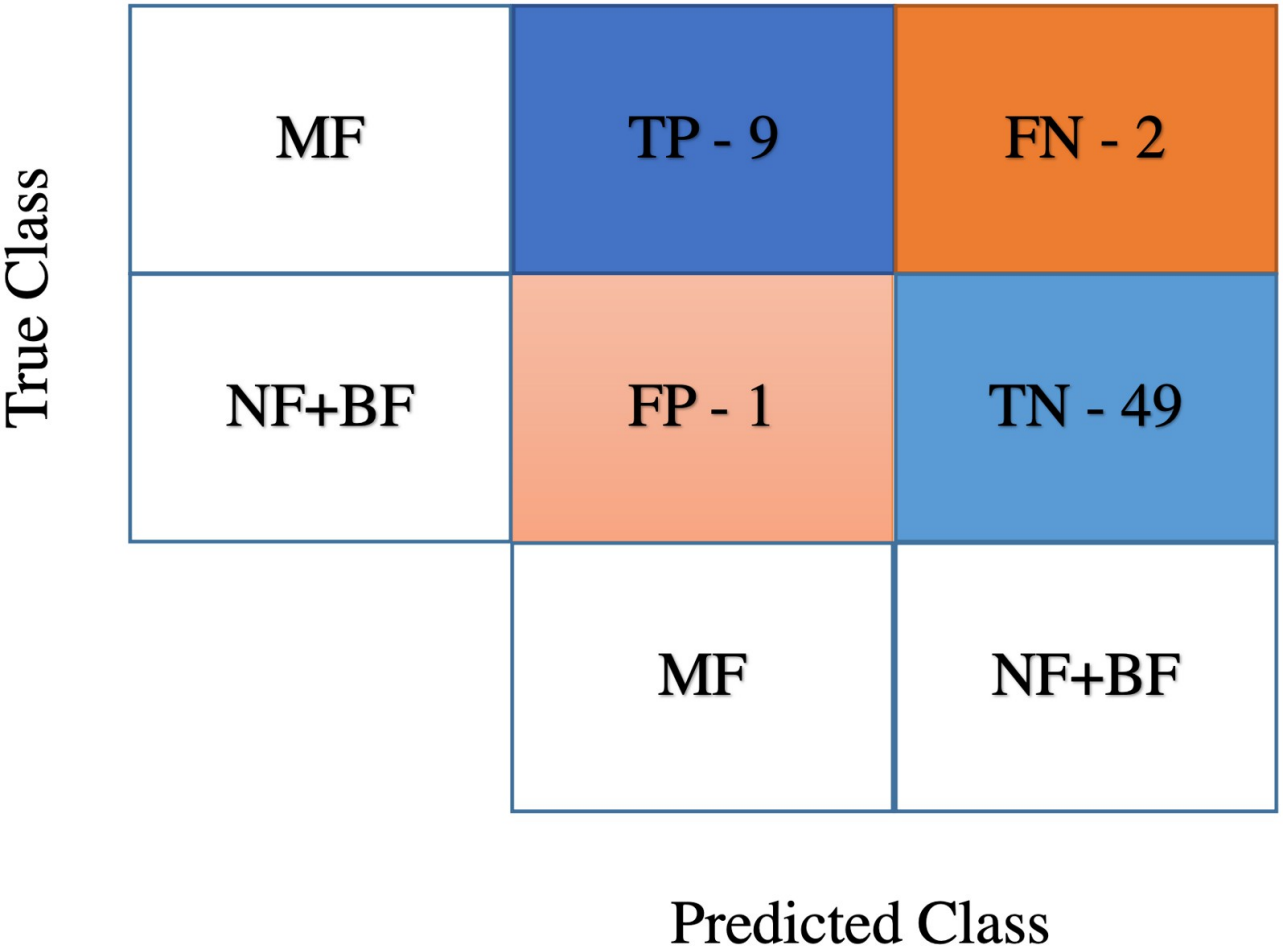
Confusion matrix obtained from the non-parametric thresholding method.

Once the PCNN applied to the images and their segmented outcomes were verified with the gold standard images, the non-parametric thresholding was applied to classify the breast patterns. In more detail, the boxplot has been examined to classify the NF+BF and MF breast patterns. Here the images for NF+BF and MF exhibit almost similar properties (mean of NF+BF breast’s *Q*_1_ ≈ 328 and MF breast’s *Q*_1_ ≈ 280, mean of NF+BF breast’s *Q*_3_ ≈ 2209 and MF breast’s *Q*_3_ ≈ 1948). Hence, the *Q*_1_ and *Q*_3_ of both types cover a broad and quantitative range of the image data and cannot differentiate breasts with these distributions. To overcome this, a 2*D* Gaussian smoothing kernel with standard deviation (*σ*) of 30 has been heuristically selected to enhance certain areas of the images using a replication padding process. Then, the boxplot has been re-examined for the breast images, illustrating difference in mean of *Q*_1_ ≈ 770 for NF+BF and *Q*_1_ ≈ 670 for MF breast types, whereas *Q*_3_ ≈ 1600 for NF+BF and *Q*_3_ ≈ 1500 for MF shows similar variability for both types of images. Based on the boxplot, quartile threshold has been selected by averaging *Q*_3_ for both breast types. The 1690 value has been found as the optimal threshold for beast identification purpose and is shown in Fig 7. Fig 7 shows all of the 61 breasts boxplots including NF, BF and MF types, where the *x*-axis determines the number of examined breasts and *y*-axis determines the peak intensity (arbitrary units) of the corresponding breast images. The threshold has been defined by a red dotted line, if the median (*Q*_2_) of each breast pattern lies beyond the threshold, then the breast is identified as MF class and the median of those breasts that lies under the threshold is considered as NF+BF breast class. The outcome of this lesion detection has been analysed via confusion matrix shown in Fig 8.

The detection performance of MF and NF+BF patterns has been validated by the true positive (TP), false negative (FN), false positive (FP), and true negative (TN) measures. The standard for the meta-analyses have been categorised as the actual output where MF patterns defined as class 1 (positive) and NF+ BF defined as class 2 (negative). The predicted output has been defined by the proposed method which determined the number of instances that are correctly detected and the instances that are wrongly detected as lesions among the total number of examined patterns and then class performance has been measured. Fig 8 shows the prediction outcomes, the true-positive of 81.82% and true-negative of 98% has been achieved by the proposed method for identifying the breast patterns from the MammoWave dataset.

## Discussion and conclusion

The proposed breast lesion detection from the non-ionizing MammoWave system aims to address three main tasks: optimal conductivity level identification for microwave image reconstruction, localisation of potential lesions, and subsequently, the detection of breast category (NF+BF vs MF). Here, the microwave images are formed by applying Huygens Principle, resulting in intensity maps representing the homogeneity of the dielectric properties of the breast tissues under test.

MammoWave images are maximum intensity projection coronal maps (2D) of the whole 3D volume of the breast. Radiologists use a quadrant representation to describe an inclusion’s (i.e lesion) location. A single breast can be divided into four quadrants: upper outer, upper inner, lower outer, and lower inner by two perpendicular planes intersected at the nipple. This representation partially overcomes lesion position mismatches introduced when using different devices. In this study, to further reduce such eventual mismatches, we performed the localization comparison (between the localization given by the radiologist using the gold standard information and the localization obtained using MammoWave) considering the breast divided only in two zones (upper and lower).

Understanding the difference between breast microwave images with and without lesions is a challenging task. For this purpose, adaptive segmentation has been performed employing pulse coupled neural network. The proposed framework has been tested empirically on 61 microwave breast images. Additionally, a non-parametric thresholding technique was modelled to differentiate between breasts with malignant finding and breasts without radiological findings or with benign finding. The presented result shows the adaptability of the proposed method to successfully capture the respective lesion’s location, identified in terms of upper/lower zone. All the locations of the lesions found matches with the radiologist’s location findings. The Box whisker plot has been used along with thresholding, achieving 81.82% sensitivity in MF detection and a specificity of 98%. The sensitivity value is in agreement both with other MammoWave analysis and with [35, 36], where the Maria system has been used (Maria system (Micrima Ltd, UK) uses an array of 60 antennas and a matching liquid to perform the radar approach).

A limitation of this study is that the pre-menstrual information of the subjects was not considered. Moreover, while performing the microwave image acquisition, some artifacts may occur due to unusual and abrupt movements of patients during their examination. Thus, elimination of these artifacts will be subsequently considered to improve performance. Also, it should be pointed out that the values of the sensitivity in MF detection and of the specificity have been experimented and validated with a limited dataset (61 breasts); performance may vary when a larger amount of data will be considered.

Further a greater number of patients’ will be engaged in the clinical study and different parameters (i.e., breast density and/or size, lesion size) will be adapted to the system for multi-level learning purpose, with the aim of improving clinical evidence on the use of MammoWave in the breast screening pathway.
